# Experience of a demand-side subsidy scheme for residential long-term care: perspectives of elderly and their carers

**DOI:** 10.1186/s12877-022-03692-2

**Published:** 2023-01-07

**Authors:** Carrie Ho-Kwan Yam, Eng-Kiong Yeoh, Eliza Lai-Yi Wong, Angel Hor-Yan Lai, Ethan Ming-Yin Ip, Tsz-Yu Chow, Kailu Wang

**Affiliations:** 1 Centre for Health Systems and Policy Research, The JC School of Public Health and Primary Care, The Chinese University of Hong Kong, Room 418, School of Public Health, Prince of Wales Hospital, Shatin, New Territories, Hong Kong, People’s Republic of China; 2 Department of Applied Social Sciences, Faculty of Health and Social Sciences, The Hong Kong Polytechnic University, Hong Kong, People’s Republic of China

**Keywords:** Residential care services, Demand side subsidy, Voucher, Features, Choice, Flexibility, Access

## Abstract

**Background:**

Vouchers, which are demand-side subsidies to targeted groups, are a type of consumer-led near-cash social transfer for specified benefits that have been used in education, health and other sectors. To provide better access to residential care services and an additional choice for elderly people in need, a novel means-tested residential care service voucher has been introduced in Hong Kong for elderly people to purchase places in the private sector to enable consumer-directed care. The objectives of this paper are to analyze the perspectives of voucher users and their carers toward the voucher scheme and to identify key elements in the design that will contribute to meeting the scheme’s objectives.

**Methods:**

An exploratory sequential mixed method design was adopted with initial explorative qualitative data collection of the perspectives of elderly people and their carers (Phase 1), which informed the design of the subsequent questionnaire survey (Phase 2). Thirty carers in 5 focus groups and 20 individual interviews with elderly people were conducted between April and May 2018. A total of 401 respondents (373 carers and 28 elderly people) completed the survey questionnaire. Findings from both phases were integrated both narratively and via a joint display.

**Results:**

Five key themes summarized the features in two main elements of the design and implementation of the voucher scheme: awareness, meaning that inadequate knowledge and understanding of voucher schemes hinder participation; service needs and types, indicating that the urgent need for residential care services is the key reason for participation; shared responsibility, meaning that a high copayment level discourages participation; choice and flexibility, reflecting appreciation of the additional choices provided by voucher schemes although the availability of residential care beds limits choices; and service quality, indicating mixed perceptions of service quality and the impact of the voucher scheme. Voucher users believe that the voucher scheme is more helpful for relieving the financial burden (98.7%), reducing carers’ stress (97.0%) and reducing the waiting time for subsidized homes for elderly people (89.0%) than for increasing choice and flexibility (78.1%) and improving service quality (62.1%).

**Conclusions:**

This study demonstrates how the design of a voucher scheme affects its take-up by targeted beneficiaries. When a voucher scheme is implemented in a long-term care system, it must consider the congruence with existing policies in long-term care provision and financing. The voucher scheme in Hong Kong has been able to generate the utilization of nonsubsidized places in homes for elderly people that were underutilized, but its effectiveness is limited by inadequate knowledge and understanding of the voucher scheme and the availability of residential care places. Giving the purchasing power and choice of providers to beneficiaries has the potential to enhance the quality of services, which will contribute to meeting the objectives. The study findings carry significant implications for long-term care policies and provide insights into the key features of the voucher scheme for residential care services and how to best design and implement a voucher scheme for elderly people in the context of policy objectives and a long-term care policy.

## Background

The demand for long-term care is increasing rapidly around the world due to the aging population and rising levels of multimorbidity as the leading cause of disability [1]. In Hong Kong, the elderly population (aged 65 and over) is projected to increase from 20.5% in 2021 to 27.6% in 2031 [2, 3]. Traditionally, informal care by family members has played a key role in providing assistance with basic self-care, mobility and household tasks and enabling elderly people to stay in the community [4, 5]. However, family support has become less prevalent due to population trends in aging, lower marriage and fertility rates, and higher divorce rates [6]. The demand for formal long-term care, such as residential care services, has been increasing. Hong Kong has a higher institutionalization rate (nearly 7% in 2009) than many other developed countries, which might be partly due to the inadequacy of community services [7]. Government spending on elderly people for residential care, community care and support, and transitional care has been gradually increasing from 10.5% of the overall social care budget in 2011–12 to 11.8% in 2016–17 and to 13.6% in 2021–22, reflecting an overall increasing trend in long-term care expenditure in Hong Kong [8–10]. These demographic and epidemiological shifts call for innovative ways to finance and deliver long-term care. Policy-makers have made it a priority to contain costs and provide affordable and sustainable long-term care services [4, 7, 11].

### Residential long-term care in Hong Kong

**Fig. 1:**
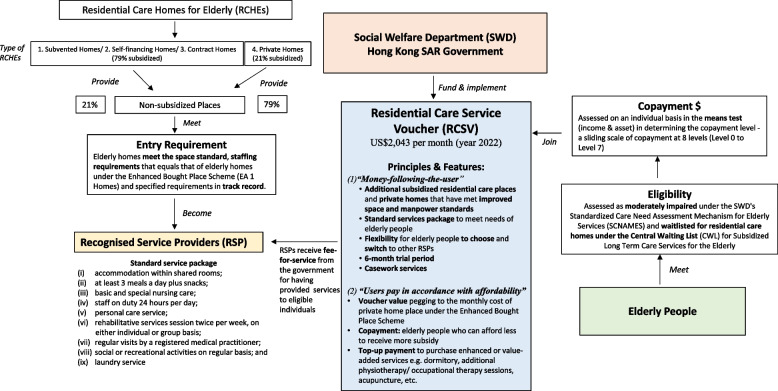
The Residential Care Service Voucher in Hong Kong

The provision of residential care services in Hong Kong is largely a publicly funded model. The government provides significant financial subsidies to nongovernmental organizations (NGOs) or private organizations to operate subsidized places in homes for elderly people. There are four types of residential care homes for elderly people (RCHEs; Fig. 1). Subvented homes are operated by NGOs that receive government funding that subsidizes 90% of the operating costs. Contract homes are operated by NGOs or private for-profit organizations that hold government contracts to operate subsidized RCHEs. In the private market, NGOs also operate not-for-profit self-financing homes to cater to more affluent elderly people seeking high-quality residential care. Private homes operated by private for-profit organizations dominate the RCHE market, accounting for 75.4% of all RCHEs [12]. The growth of private homes has occurred in response to the unmet needs of the elderly who may face waiting times of 3–4 years for a place in public sector homes or for those who can afford higher-quality homes. The government offered cash transfers to low-income elderly to be able to afford a place in private for-profit homes under the Comprehensive Social Security Assistance (CSSA) Scheme [7, 13]. However, this had an unintended effect on quality because the average cash transfers were very modest at approximately US$1400 per month, and private homes had little incentive beyond meeting the minimum space and licensing standards.

To meet the demand pressures and to improve the quality of private RCHEs, in 1998, the government introduced the Enhanced Bought Place Scheme (EBPS) and started to purchase residential care places that met a high standard of space and manpower provision. In addition, the government implemented a Standardized Care Need Assessment Mechanism for Elderly Services (SCNAMES) to better assess people’ needs and determine the care provision to be assigned [14]. In 2003, the government created a Central Waiting List for public subsidized placements of elderly people assessed by SCNAMES.

In 2022, the monthly payment for a public subsidized place was $263 (USD) per month without regard to financial means. In comparison, the monthly payment for a private nonsubsidized place was much more expensive, ranging from $573–2676 (USD) per month [15]. Compared to private subsidized homes, the service quality of public subsidized homes is set by the government at a relatively higher standard of space and staff requirements and is funded at a higher level of operating costs [4, 6, 13, 16, 17]. This has created a segmentation of high-quality, low-cost publicly subsidized residential care places and generally lower-quality, high-cost private residential care places serving a common pool of elderly people who need residential care. In this policy environment, elderly people prefer to wait for a publicly subsidized place. As of August 2022, there were 18,128 applicants on the waiting list for placement in 29,143 subsidized residential care places, with a waiting time of 41 months for public subsidized places and 6 months for subsidized places in private homes under the EBPS [18]. It was projected that some 64,000 subsidized residential care places would be required by elderly people in 2030.

### Demand-side subsidy

Demand-side subsidies are a demand-side financing tool that was first used in developing countries to improve service utilization by underprivileged groups by giving them subsidies to purchase services from designated providers [19]. The subsidy can take the form of a conditional cash transfer, tax rebate, or voucher that is a token that can be exchanged for services. Typically, vouchers are funded either by a donor or the government and are distributed to the target group in paper or electronic form for specified services from private service providers enrolled in the program. In addition to reducing the financial hardship of service users in accessing services [20–24], vouchers can be designed to facilitate consumer-directed care by giving greater autonomy, choice and control to the recipients to select care providers and services that best fit their needs and can improve service efficiency by introducing a choice of private sector providers in the care market and creating competition [21, 22, 25, 26]. There is a wealth of literature on health vouchers in developing countries, especially regarding immunization and maternal and child health. Evidence for the effectiveness of the vouchers has been mixed and is affected not only by the design and the context but also by how they are implemented. Although voucher schemes have been found to be more successful in specified health preventive services, particularly those that are well defined and time limited [21, 23–25, 27], there is very limited literature on health care voucher schemes in developed economies, particularly for long-term care. This gap is particularly apparent in long-term care in Asia, where there is a predominant mixed public–private economic model in financing health care.

### Residential care service vouchers in Hong Kong

In March 2017, the Social Welfare Department (SWD) of the Hong Kong Special Administrative Region government introduced a novel means-tested pilot scheme for residential care services vouchers to enable consumer-directed care in the purchase of private sector places. This voucher scheme will be regularized in 2022–23. The targeted beneficiaries of the voucher scheme are elderly people who have been assessed by SCNAMES as moderately impaired and are on the central waiting list.

There are several features in the design of the voucher scheme (Fig. 1). First, the demand-side “money-following-the-user” principle of the scheme provides greater flexibility and choice for elderly people to purchase nonsubsidized residential care places from contract/subvented homes, self-financing homes and private homes. Voucher users can freely choose and switch between participating homes for elderly people under the voucher scheme. Second, the principle that “users pay in accordance with affordability” allows elderly people who can afford less to receive a larger subsidy from the government. The current voucher value is $2043 per month (USD), which is adjusted annually according to inflation. Voucher applicants are means-tested on an individual basis according to elderly people’ income and assets to determine copayment levels ranging from Level 0–7 [28]. Those assessed at copayment level 0, including those who meet the CSSA eligibility requirements, do not pay a copayment. Currently, voucher users pay between $204 (copayment level 1) and $1532 (copayment level 7) compared to a no-mean-tested payment ($263) for subsidized public homes for elderly people. Voucher users can also contribute up to 150% of the voucher value to purchase enhanced services, such as additional physiotherapy/occupational therapy sessions to meet their needs. Third, there is a 6-month trial period for first-time voucher users to adapt to life in the participating homes for elderly people, during which time they can withdraw from the scheme and be reinstated on the central waiting list. Other features of the voucher scheme include casework services that are provided by social workers to assist users in understanding the scheme, choosing suitable services, offering follow-up support after admission, and conducting regular visits. To ensure service quality under the voucher scheme, participating homes for elderly people need to meet the space and staffing requirements set by the government and to provide “a standard service package” including meals, basic and special nursing care, rehabilitative services sessions per week, and social or recreational activities. As of November 2019, there were 1617 cumulative voucher users [29]. In July 2021, among 671 homes for elderly people that provided private residential care services (including homes that were not previously recognized as being able to meet the standards required for participation), 23.4% (157) joined the voucher scheme. A total of 18.5% of participating homes for elderly people were subvented/contract homes, while the remaining majority were self-financed or private homes [30–32].

The objectives of this paper are to analyze the perspectives of voucher users and their carers toward residential care service vouchers, including their perceived needs, choices and experience, and to identify key elements in the design that will contribute to meeting the objectives of the voucher scheme. Evidence generated from this study will inform long-term care systems in other economies about how the design and implementation of a voucher scheme can improve access to residential care services and enable choice and flexibility to meet the needs of elderly people.

## Methods

An exploratory sequential mixed method design was adopted with initial explorative qualitative data collection of the perspectives of voucher users and nonusers and their carers (Phase 1), which informed the design of the subsequent questionnaire survey (Phase 2) [33]. Voucher users refer to all users who have ever used a voucher, including current users residing in participating homes for elderly people and users who withdrew and left the home after having used the voucher (with an admission record for a participating home for elderly people). Nonusers refer to elderly people who did not apply for the scheme upon invitation by the SWD and those who applied for the scheme but withdrew before actual admission to a participating home. The qualitative data were first collected and analyzed, and the themes that emerged were used to develop the survey instrument to better understand the perspectives and experiences of elderly people and their carers regarding the voucher scheme. The reason for collecting qualitative data was twofold. First, the voucher scheme was a novel policy with no previous experiences to build on; second, there is little guiding theory in the contextual application and implementation of a voucher policy in developed economies. As such, an exploratory approach was first needed to direct the design of the quantitative phase. Findings from both phases were integrated narratively and via a joint display. Ethical approval was obtained for the study from the Survey and Behavioral Research Ethics Committee at the Chinese University of Hong Kong. Written consent was obtained from all respondents after a clear explanation of the study objectives and to ensure data confidentiality.

### Qualitative strand (phase 1)

The targets of the qualitative interviews were voucher users and nonusers and their carers. They were stratified by whether they were recipients of the government’s Comprehensive Social Security Assistance, the type of home for elderly people and the copayment level. A purposive sampling strategy was used to select participants for the discussions/interviews. Data were collected in focus group discussions with carers of voucher users and nonusers, and individual face-to-face interviews with users. Individual interviews were employed for voucher users instead of focus groups due to the mobility limitations of elderly people. The duration of each focus group discussion and individual interview was approximately 60 and 30 minutes, respectively. Interviews were conducted in the participants’ mother tongue (in Cantonese) by a trained researcher. Discussions and interviews were continued until issues were judged to be saturated theoretically and no new relevant data emerged [34].

A discussion guide of open-ended questions was developed based on the literature review, the objectives and design of the voucher scheme and the research team’s knowledge and experiences in elderly care. The discussion guide for carers covered (a) understanding and attitudes toward the voucher, including its design and implementation, (b) the type of services the care recipients received and whether the services met their care needs, and (c) the impact of the voucher scheme. The discussion guide for elderly participants was similar to that for carers with an additional focus on their experience staying in the homes for elderly people.

The focus groups and interviews were audio-recorded with the participants’ consent and transcribed verbatim. The transcripts were analyzed for recurring themes using NVivo software. A thematic analysis was adopted. The transcript was read by one researcher to identify possible broad themes. If these emergent themes occurred repeatedly across and within the transcripts, they were noted as recurrent themes. A second researcher read the transcripts and independently generated emergent and recurrent themes. Subsequently, the two researchers discussed and agreed on emergent themes and then examined the transcripts for any connections among the recurrent themes. The researchers discussed with the principal investigator of this study to agree on the themes. Related recurrent themes were organized under a master theme. Interpretations of the themes are illustrated by extracts from the transcripts.

### Qualitative data-informed quantitative design (phase 2)

The questionnaire was first designed based on the literature and themes identified from the qualitative study, reviewed and commented on by the team, and finally pilot tested. The questionnaire started with questions on the respondents’ awareness of the voucher. This was followed by questions related to their attitudes and satisfaction with the design of the voucher scheme, which included the “standard service package”, copayment mechanism, and support from social workers. Their views on the impact of voucher schemes in reducing the waiting time for residential care services, providing choice and flexibility, and improving the service quality of service providers were also sought.

Elderly people were recruited randomly from the list of voucher users and nonusers of the administrative database maintained by SWD as of October 31, 2018. The survey was conducted in face-to-face interviews by trained interviewers with a structured questionnaire in Chinese at a place convenient to the respondents. Carers were invited to answer as a proxy if the elderly individual could not respond due to cognitive impairment/dementia, hearing impairment, or difficulties with speech. Consent was obtained before conducting the interview, and each interview lasted approximately 30 minutes. Data were analyzed using R software. Each elderly individual was assigned a unique individual identifier number to ensure confidentiality. Descriptive statistics on the attitudes and experiences of voucher schemes are presented.

## Results

Five focus groups with 30 carers of voucher and nonvoucher users and 20 individual interviews with elderly people from the 4 different types of residential care homes were conducted from April–May 2018 (Table 1). For the questionnaire survey, a total of 401 elderly people/carers (373 carers and 28 elderly people) were interviewed from January–June 2019, with a response rate of 72.2%. The number of voucher users was higher than that of nonusers due to the low rate of willingness of nonusers to participate. The results of voucher users and nonusers are presented separately. Among 301 (75.1%) voucher users, 71.4% were female, and more than 80% were aged 80 years old or above (Table 2). Among 100 nonusers, 62.0% were female, and more than 70% were aged 80 years old or above. Most of the elderly people (95% for both users and nonusers) received public allowances from the government.

**Table 1:** Number of focus groups/ interviews per stakeholder group

Group	Date	No of focus group/ interviews	Age range	Government social security assistance	Types of participating homes for elderly people
Carers of voucher users (focus group)	18/4/2018 20/4/2018 24/4/2018	3 groups including 18 carers	76–105	9 ex-recipients; 11 non-recipients	6 contract homes; 2 self-financing homes, 10 private homes
Carers of nonvoucher users i.e. elderly who had withdrawn the voucher scheme before using the voucher (focus group)	11/5/2018 14/5/2018	2 groups including 11 carers	81–99	6 ex-recipients; 11 non-recipients	NA
Elderly (Individual interview)	27/4/2018–18/5/2018	20 elderly	65–96	9 ex-recipients; 11 non-recipients	10 contract homes; 1 self-financing homes, 9 private homes

**Table 2:** Demographic characteristics of the elderly respondents in the questionnaire survey (*n* = 401)

Demographic characteristics	Total (***n*** = 401)	Voucher user (***n*** = 301)	Non voucher users (***n*** = 100)
*Gender*			
Male	124 (30.9)	86 (28.6)	38 (38.0)
Female	277 (69.1)	215 (71.4)	62 (62.0)
*Age*			
≤ 70 years old	19 (4.7)	10 (3.3)	9 (9.0)
71–80 years old	50 (12.5)	36 (12.0)	14 (14.0)
81–90 years old	204 (50.9)	152 (50.5)	52 (52.0)
> 90 years old	128 (31.9)	103 (34.2)	25 (25.0)
*Marital status*			
Never married	17 (4.2)	12 (4.0)	5 (5.0)
Widowed	253 (63.1)	200 (66.4)	53 (53.0)
Divorced	16 (4.0)	13 (4.3)	3 (3.0)
Married	108 (26.9)	71 (23.6)	37 (37.0)
Separated	7 (1.7)	5 (1.7)	2 (2.0)
*Current living status*			
Live alone	9 (2.2)	0 (0)	9 (9.0)
Live with family/ others	48 (12)	4 (1.3)	44 (44.0)
Live in institution e.g. hospital, convalescent hospital	1 (0.2)	0 (0)	1 (1.0)
Live in old age home	343 (85.5)	297 (98.7)	46 (46.0)
*Living district*			
Hong Kong Islands	33 (8.2)	22 (7.3)	11 (11.0)
Kowloon	240 (59.9)	192 (63.8)	48 (48.0)
New Territories	128 (31.9)	87 (28.9)	41 (41.0)
*Sources of income – Can choose more than one options*			
Pension	11 (2.7)	5 (1.7)	6 (6.0)
Financial support from children/ other relatives	18 (4.5)	10 (3.3)	8 (8.0)
Comprehensive Social Security Assistance	28 (7.0)	1 (0.3)	27 (27.0)
Disability allowance	20 (5.0)	14 (4.7)	6 (6.0)
Old Age Allowance	335 (83.5)	272 (90.4)	63 (63.0)
Other sources of income e.g. rental income	2 (0.5)	0 (0)	2 (2.0)
No income	8 (2.0)	8 (2.7)	0 (0)
Don’t know	3 (0.7)	3 (1.0)	0 (0)

Five key themes summarized the features in two main elements of the design and implementation of residential care service vouchers in the joint display shown in Table 3:

- Awareness: Inadequate knowledge and understanding of voucher schemes that hinder participation.
- Service needs and types: The urgent need for residential care services is a key reason for participation.
- Shared responsibility: A high copayment level discourages participation.
- Choice and flexibility: Appreciation of the additional choices provided by the voucher scheme; however, the availability of residential care beds limits choice.
- Service quality: Perceptions of service quality and the impact of the voucher scheme are mixed.

**Table 3:** Key themes identified from the focus group discussions/ individual interviews and questionnaire survey

Theme	Focus groups/ Individual interviews	Questionnaire survey
**(i) Awareness of voucher scheme**	– Some elderly people and carers expressed limited knowledge and understanding of the voucher scheme – The roles of social workers (both social workers and caseworkers) are pivotal during their applications – The copayment mechanism was complicated and difficult for the elderly people and their carers to understand by only reading the invitation letter and the introduction leaflet	– 10% of the nonusers revealed that insufficient knowledge and understanding of the scheme was the reason for no-participation in the voucher scheme – A higher proportion of users (68.1%) than nonusers (45.0%) thought that the voucher scheme information was sufficient – Users generally had a better understanding of the pilot scheme than nonusers
**(ii) Service needs and types**	– Carers believed that the immediate need for residential care services emerging from sudden changes in the health condition of elderly people was the main reason for joining the voucher scheme – Many carers noted that the voucher scheme allows elderly people to be placed in homes in a substantially shorter period of time compared to the waiting lists for subsidized homes – Carers of elderly people who withdrew from the voucher scheme no longer indicated immediate needs. A number of nonusers did not accept the copayment level, and some stated that there were no places available in their preferred homes	– 45.8% of users joined the voucher scheme due to an urgent need for residential care services – 39.5% joined because they believed that the scheme could shorten their waiting time for subsidized homes for elderly people – 34.0% of non-users did not join the voucher scheme because the elderly people did not have an immediate need for residential care services – 87.8% of voucher users and 79.0% of nonusers agreed that the service package under the voucher value could meet the needs of the elderly people
**(iii) Shared responsibility**	– A few of the carers agreed in principle with the idea of shared responsibility – Many carers felt that unless the copayment was equal to or lower than the non-means-tested standard copayment fees charged for all government-funded places in the different types of homes allocated from the central waiting list, elderly people would have less incentive to participate – Some further noted that the voucher scheme was not particularly attractive for elderly people who were assessed at copayment level 7 (i.e., co-pay of $1532) as the amount of subsidy was relatively low. – There were concerns among carers that the possibility of an increase in fees of the homes for elderly people in the future might exceed the adjustment in voucher value	– 66.8% of voucher users were willing to make top-up payments to purchase enhanced or value-added services in addition to the standard service package – 86.7% of voucher users and 80.0% of nonusers agreed that in the means test, the applicants should only be assessed on their personal income and asset to determine the copayment level and not that of their households – 72.8% of voucher users and 57.0% of nonusers (57.0%) thought the copayment mechanism was suitable – The majority of vouchers users who agreed with the means test and copayment mechanism were at copayment level 0 (without copay)
**(iv) Choice and flexibility**	– Valued the choice and flexibility of the voucher scheme because it provided additional subsidized residential care places on top of the current places allocated from the central waiting list and the enhanced bought places scheme. However, some carers reported that their choice was limited by the number of homes participating in the voucher scheme and the limited availability of bed places in their preferred homes	– 78.1% of elderly people and their carers thought the voucher scheme was helpful in increasing their choice and flexibility in residential care services, while 14.6% thought it was not helpful – Those who thought it was helpful was mainly those who were currently already in homes for elderly people and those with shorter waiting times on the central waiting list as of their date of admission to a participating home under the voucher scheme (*p* value < 0.05) – 11% of the non-users indicated that there was no available bed place in their preferred participating homes for the elderly people; and 7% stated that they did not prefer the participating homes for elderly people on the list as their reason for nonparticipation
**(v) Service quality**	– Many users and their carers were generally satisfied with the service quality of participating homes for the elderly people, despite dissatisfactions in some areas, such as manpower, hygiene, environment and the attitude of the staff in the homes. They added that the entry requirement set by the government on staff and space for the participating homes could ensure quality – For carers of nonusers, they felt that the environment and service quality among participating homes varied, particularly in private homes. However, they thought that the idea of money-following-the-user could encourage participating homes for elderly people to improve their service quality to remain competitive and to attract more admissions, since if the participating homes did not provide service high-quality services, voucher users could select or switch other homes with better service quality – Some carers believed the casework service by the government would have a positive effect on the service quality of participating homes for the elderly, and could indirectly improve their service quality through regular checking by caseworkers	– 93.7% of voucher users agreed that the support received from caseworkers was sufficient – 62.1% of voucher users and their carers agreed that the voucher scheme was helpful to improve the service quality of homes for elderly people (60% were already living in these homes before participating in the voucher scheme) – However, voucher users thought that the voucher scheme was more helpful in relieving their financial burden (98.7%), reducing carers’ stress (97.0%) and the waiting time for subsidized homes (89.0%), increasing choice and flexibility (62.1%), and improving service quality (62.1%)

### Theme 1: awareness of voucher scheme

#### Qualitative study

Some elderly people and carers expressed limited knowledge and understanding of the voucher scheme when they received invitation letters from the government. The copayment mechanism of the voucher scheme was complicated and difficult for them to understand by only reading the invitation letter and the introduction leaflet. The referral and coordination of social workers in the centers for elderly people and explanations by caseworkers played a pivotal role in their decision to apply for the voucher scheme.

“We had no prior knowledge about that, and we totally had no idea what it was when we received the invitation letter. After that, we called the voucher office for enquiry and the caseworkers explained this new scheme to us….” (C203 Carer of user).

“In general, we were able to understand most of the content in the invitation letter. When there were areas which were difficult to understand, the responsible worker [social worker] who has been following my mom’s case would call the voucher office to enquire. We could call the office for more information too.” (C204 Carer of user).

“I only understood part of the voucher scheme. For the copayment, I thought the self-occupied property would not be counted as an asset as our family is living here. We are not renting it out…

We dared not to apply at first, and we only applied for the scheme when we received the second invitation letter. It turned out we had to copay around $1200. If we knew we had to copay this amount, we would not have applied for that in the first place.” (C503 Carer of nonuser).

Some of the elderly participants also reported that they were confused by the various government schemes for residential care services, such as subsidized homes under the central waiting list and residential care places under the enhanced bought places scheme. A few even did not know which scheme they were participating in.

“I haven’t heard of the voucher scheme…I know the government is paying for my bed place here.” (E12 Voucher user).

#### Quantitative study

Ten percent of the nonusers revealed that insufficient knowledge and understanding of the scheme was the reason for nonparticipation in the voucher scheme (Table 4). A higher proportion of users (68.1%) than nonusers (45.0%) thought that the voucher scheme information was sufficient. Those who thought the information was insufficient wanted more information about the design of the voucher scheme (32.0%) and eligibility for application (35.2%). Regarding understanding of the scheme, most respondents responded correctly regarding the existence of means-test mechanisms to determine the copayment level (88.5%), the opt-out mechanism from the central waiting list for long-term care services (82.5%), and the flexibility to switch to other homes for elderly people (79.8%). There were relatively lower levels of understanding of the top-up payments and eligibility for social security assistance. Users generally had a better understanding of the voucher scheme than nonusers (*P* value < 0.05).

**Table 4:** Attitudes towards the voucher scheme (*n* = 401)

Variables	Total (***n*** = 401)	Voucher user (***n*** = 301)	Non voucher users (***n*** = 100)
Understanding of the Pilot Scheme - % of people answered correctly			
(1) There is a mean-test to determine the co-payment mechanism	355 (88.5)	289 (96.0)	66 (66.0)
(2) Voucher holders need to opt out from the Central Waiting List of the Long Term Care Services Delivery System after the trial period	331 (82.5)	278 (92.4)	53 (53.0)
(3) Voucher holder can switch to other participating homes for the elderly people	320 (79.8)	267 (88.7)	53 (53.0)
(4) Voucher holders can make top-up payments to purchase enhanced or value added services that are outside the standard service package	277 (69.1)	219 (72.8)	58 (58.0)
(5) Voucher holder is not eligible for Comprehensive Social Security Assistance Scheme	269 (67.1)	226 (75.1)	43 (43.0)
For users only Reasons for joining voucher scheme (*n* = 301) – Can choose more than one option			
(1) Making good use of the subsidy	193 (64.1)	193 (64.1)	–
(2) Urgent need for residential care services	138 (45.8)	138 (45.8)	–
(3) Shorten waiting time on the Central Waiting List of the Long Term Care Services Delivery System	119 (39.5)	119 (39.5)	–
(4) Increase choice and flexibility	50 (16.6)	50 (16.6)	–
(5) Better quality of participating homes for the elderly people	40 (13.3)	40 (13.3)	–
(6) Can purchase top-up services to increase quality	2 (0.7)	2 (0.7)	–
Whether willing to make top-up payments to purchase enhanced or value-added services other than the standard service package or not (*n* = 301)			
Yes	201 (66.8)	201 (66.8)	–
No	90 (29.9)	90 (29.9)	–
Don’t know	10 (3.3)	10 (3.3)	–
For non-users only Reasons for not joining or withdrawing from the voucher scheme (*n* = 100) – Can choose more than one option			
(1) The elderly does not have an immediate need for residential care services	34 (34.0)	–	34 (34.0)
(2) The elderly prefers to wait for subsidized places on the Central Waiting List of the Long Term Care Services Delivery System	25 (25.0)	–	25 (25.0)
(3) The elderly does not adapt/ suitable/ want to live in residential care homes	13 (13.0)	–	13 (13.0)
(4) There is no available place in his/her preferred participating homes for the elderly	11 (11.0)	–	11 (11.0)
(5) Insufficient knowledge and understanding about the scheme	10 (10.0)	–	10 (10.0)
(6) The elderly does not agree with the copayment mechanism	10 (10.0)	–	10 (10.0)
(7) The elderly has already placed at a subsidized residential care homes	9 (9.0)	–	9 (9.0)
(8) Expect the elderly will place to a subsidized residential care homes soon	8 (8.0)	–	8 (8.0)
(9) There is no preferred service provider in the list of the participating homes for the elderly	7 (7.0)	–	7 (7.0)
(10) Preferred residential care homes do not accept the voucher	5 (5.0)	–	5 (5.0)
(11) The elderly does not want to leave the comprehensive social security assistance	4 (4.0)	–	4 (4.0)
Whether sufficient information provided on the scheme			
Sufficient	250 (62.3)	205 (68.1)	45 (45.0)
Fair	47 (11.7)	35 (11.6)	12 (12.0)
Not Sufficient	79 (19.7)	48 (15.9)	31 (31.0)
Don’t know	25 (6.2)	13 (4.3)	12 (12.0)
What kind of information should be enhanced (for those who thought the information “fair” or “not sufficient”) – Can choose more than one options			
Eligibility for application	44 (35.2)	27 (32.9)	17 (39.5)
The design e.g. mean tests, voucher value, copayment, top-up payment and trial period	40 (32.0)	26 (31.7)	14 (32.6)
Channels to disseminate the list of participating homes for the elderly people	26 (20.8)	19 (23.2)	7 (16.3)
The objectives of the voucher scheme	18 (14.4)	12 (14.6)	6 (14.0)
The scope of service	7 (5.6)	4 (4.9)	3 (7.0)
Whether the “standard service package” under the voucher value for individual voucher holders can meet the elderly people’s needs			
Yes	343 (85.5)	264 (87.7)	79 (79.0)
No	49 (12.2)	35 (11.6)	14 (14.0)
Don’t know	9 (2.2)	2 (0.7)	7 (7.0)
Whether the voucher applicants should be assessed on an individual basis in the means test taking into account both income and asset to determine the copayment level			
Agree	341 (85.0)	261 (86.7)	80 (80.0)
Disagree	48 (12.0)	32 (10.6)	16 (16.0)
Don’t know	12 (3.0)	8 (2.7)	4 (4.0)
Whether the current copayment mechanism (including number of copayment levels, income and asset limit for each level) is suitable			
Agree	276 (68.8)	219 (72.8)	57 (57.0)
Disagree	88 (21.9)	57 (18.9)	31 (31.0)
Don’t know	37 (9.2)	25 (8.3)	12 (12.0)
Whether the support received from case workers of Residential Care Services Voucher Office is sufficient			
Agree	282 (93.7)	282 (93.7)	–
Disagree	8 (2.7)	8 (2.7)	–
Don’t know	11 (3.7)	11 (3.7)	–
For users only – Helpfulness of voucher scheme (*n* = 301)			
(1) Provide financial assistance to elderly people to obtain residential care services			
Helpful	297 (98.7)	297 (98.7)	–
Not helpful	3 (1.0)	3 (1.0)	–
Don’t know	1 (0.3)	1 (0.3)	–
(2) Reduce carers’ stress			
Helpful	292 (97.0)	292 (97.0)	–
Not helpful	7 (2.3)	7 (2.3)	–
Don’t know	2 (0.7)	2 (0.7)	–
(3) Reduce waiting time of residential care services			
Helpful	268 (89.0)	268 (89.0)	–
Not helpful	24 (8.0)	24 (8.0)	–
Don’t know	9 (3.0)	9 (3.0)	–
(4) Increase choice and flexibility in residential care services			
Helpful	235 (78.1)	235 (78.1)	–
Not helpful	44 (14.6)	44 (14.6)	–
Don’t know	22 (7.3)	22 (7.3)	–
(5) Improve service quality of participating homes for the elderly people			
Helpful	187 (62.1)	187 (62.1)	–
Not helpful	81 (26.9)	81 (26.9)	–
Don’t know	33 (11.0)	33 (11.0)	–

### Theme 2: service needs and types

#### Qualitative study

Carers believed that the immediate need for residential care services emerging from sudden changes in the health condition of elderly people was the main reason for joining the voucher scheme. Many carers noted that the voucher scheme allows elderly people to be placed in homes in a substantially shorter period of time compared to the waiting lists for subsidized homes. Carers of elderly people who withdrew from the voucher scheme no longer indicated immediate needs. A number of nonusers did not accept the copayment level, and some stated that there were no places available in their preferred homes.

“My mother had been waiting on the central waiting list for more than two years, and it was unlikely for her to be placed in subsidized home in the near future. Since she was in need of subsidized residential care services, that’s why she joined the voucher scheme.” (C204 Carer of user).

“My mother-in-law suddenly became blind because of diabetes. She was in urgent need of intensive care…A bed place in the voucher scheme could be arranged in a short period of time.” (C203 Carer of user).

Many of the voucher users and their carers found that the standard service package provided by the homes for elderly people was sufficient to meet their needs, so there was less intention to purchase enhanced services. Some of them reflected that top-up services may not be essential, such as additional exercise sessions and acupuncture. A few carers suggested adding more service types, such as counseling services, as top-up services.

“Basically, 90% of the services under the service package is enough. An additional thing could be more exercise, but it really depends on the elderly person’s mobility. Some of them can barely move.” (C402 Carer of nonuser).

“Counseling service is quite important. Elderly people could become really hysterical after living in an home for elderly people.” (C102 Carer of user).

#### Quantitative study

Among 301 users and their carers, 45.8% answered that the reason for joining the voucher scheme was due to an urgent need for residential care services, and 39.5% indicated that they believed that the scheme could shorten their waiting time for subsidized homes for elderly people. Univariate analysis showed that voucher users were more likely to be older, female, and without carers (*p* value < 0.10). They were also more likely to live in homes for elderly people before admission into the voucher scheme (*p* value< 0.05). Regarding the reason for not joining/withdrawing (*n* = 100), 34.0% responded that the elderly person did not have an immediate need for residential care services. Among them, 55.9% said that “family members/carers can take care of the elderly at home”. Most voucher users (87.8%) and nonusers (79.0%) agreed that the service package under the voucher value could meet the needs of the elderly person. Among those who disagreed (14.5%), the services they would like to have included were rehabilitation service sessions (24.1%), escort services (13.8%), personal-oriented rehabilitation/caring services (10.3%), and emotional support/counseling services (6.9%).

### Theme 3: shared responsibility

#### Qualitative study

The idea of shared responsibility based on the affordability of the copayment by service users was one of the key features of the voucher scheme to encourage the idea that “users pay in accordance with affordability”. Although a few of the carers agreed in principle with the idea of shared responsibility, many carers felt that unless the copayment was equal to or lower than the non-means-tested standard copayment fees charged for all government-funded places in the different types of homes allocated from the central waiting list, elderly people would have less incentive to participate. Some further noted that the voucher scheme was not particularly attractive for elderly people who were assessed at copayment level 7 (i.e., copay of $1532) as the amount of subsidy was relatively low. There were also concerns among carers that the possibility of an increase in fees of the homes for elderly people in the future might exceed the adjustment in voucher value, leading to a higher actual copayment by elderly people and their carers.

“If I wait for a subvented/contract home on the central waiting list, the home fee is fixed for all elderly people, regardless of their assets.” (C202 Carer of user).

“…I determined that the home fee of the participating homes for elderly people increased 10% annually since 2016. It might soon charge higher than the voucher value….” (C306 Carer of user).

#### Quantitative study

Most of the voucher users (86.7%) and nonusers (80.0%) agreed that in the means test, the applicants should only be assessed on their personal income and assets to determine the copayment level and not that of their households. A higher proportion of voucher users (72.8%) than nonusers (57.0%) thought the copayment mechanism was suitable. The majority of voucher users who agreed with the means test and copayment mechanism were at copayment level 0 (without copay). A total of 66.8% of voucher users were willing to make top-up payments to purchase enhanced or value-added services in addition to the standard service package.

### Theme 4: choice and flexibility

#### Qualitative study

Many carers valued the choice and flexibility of the voucher scheme because it provided additional subsidized residential care places on top of the current places allocated from the central waiting list and the enhanced bought places scheme. However, some carers reported that their choice was limited by the number of homes participating in the voucher scheme and the limited availability of bed places in their preferred home. Some carers preferred homes for elderly people in the vicinity of their neighborhood; however, their preferred homes did not participate in the voucher scheme. Most carers further noted that they preferred subvented or contract homes; however, there were very limited vacancies in these homes, leading to a long waiting time for participating homes. Some carers noted pressure and anxiety when searching for places in suitable homes for elderly people after the voucher was issued.

“After we applied, it was not as good as expected. I called more than 20 participating homes for elderly people, and all of them had no vacancy for the voucher scheme.” (C108 Carer of user).

“In terms of the choice of participating homes for elderly people, I think the number of participating homes for elderly people should be increased. Another important point is that there should be enough vacancies in these participating homes for elderly people. Otherwise, it is meaningless.” (C205 Carer of user).

Participants valued the flexibility of the voucher scheme, which allowed elderly people to opt out the voucher scheme after a 6-month trial period to return to the central waiting list for subsidized homes for elderly people. They also appreciated the flexibility to switch homes freely among the list of participating homes in the voucher scheme because this was not an option for those admitted through the central waiting list.

“I think it is very helpful and flexible. If you don’t like the participating homes for elderly people or the voucher scheme, you can go back to the central waiting list for subsidized homes for elderly people.” (C203 Carer of user).

#### Quantitative study

A total of 78.1% of elderly people and their carers thought the voucher scheme was helpful in increasing their choice and flexibility in residential care services, while 14.6% thought it was not helpful. Those who thought it was helpful were mainly those who were currently already in homes for elderly people and those with shorter waiting times on the central waiting list as of their date of admission to a participating home under the voucher scheme (*p* value < 0.05). Eleven percent of the nonusers indicated that there was no available bed in their preferred participating home for elderly people, and 7% stated that they did not prefer the participating homes on the list as their reason for nonparticipation.

### Theme 5: service quality

#### Qualitative study

Many users and their carers were generally satisfied with the service quality of participating homes for elderly people despite dissatisfaction in some areas, such as manpower, hygiene, environment, and the attitude of the staff in the homes. They added that the entry requirement set by the government on staff and space for the participating homes could ensure quality. The carers of nonusers felt that the environment and service quality among participating homes varied, particularly in private homes. However, they thought that the idea of money-following-the-user could encourage participating homes for elderly people to improve their service quality to remain competitive and to attract more admissions since if the participating homes did not provide high-quality service, voucher users could select or switch to other homes with better service quality.

“I think my mom’s home for elderly people achieved 80 marks already, definitely not 100 marks, but 80 is already satisfactory. She told me that sometimes the health care workers might not be able to take good care of her.” (C102 Carer of user).

“I visited many of the participating homes for elderly people from the list. Some of them with available bed places were unsatisfactory in terms of quality and environment.” (C505 Carer of nonuser).

Many elderly people and carers applauded the casework service and considered it very helpful. Some carers believed the casework service by the government would have a positive effect on the service quality of participating homes for elderly people and could indirectly improve their service quality through regular checking by caseworkers.

“When someone from the authority visits and does the checking, the staff in the participating homes for elderly people will do better and improve.” (C505 Carer of nonuser).

“If we have anything unsatisfactory with the participating home for elderly people, we can tell the caseworker. The participating home for elderly people is scared of case workers and will do better.” (Focus Group (C108 Carer of user).

#### Quantitative study

The majority of voucher users (93.7%) agreed that the support received from caseworkers was sufficient. A total of 62.1% of voucher users and their carers agreed that the voucher scheme was helpful to improve the service quality of homes for elderly people (60% were already living in these homes before participating in the voucher scheme) (Table 4). However, voucher users thought that the voucher scheme was more helpful in relieving their financial burden (98.7%), reducing carers’ stress (97.0%) and the waiting time for subsidized homes (89.0%), increasing choice and flexibility (62.1%), and improving service quality (62.1%).

## Discussion

This paper seeks to understand the perspectives and experiences of elderly people and their carers in a demand-side subsidy scheme for residential care and identifies key elements in the design and implementation of the voucher that affect take-up and could impact the objectives of the scheme. To the best of our knowledge, this is one of the first studies to analyze how the design of a voucher affects take-up by beneficiaries in a sequential mixed-method research design that quantifies the perspectives of the target population. Awareness and understanding of different government residential care schemes, particularly better appreciation of the benefits of the voucher scheme in meeting the needs of elderly people and their carers, was found to be critical to encourage participation. This was also found in a study in the United Kingdom [35, 36]. There was general understanding of the complex design of the voucher, which was enabled by caseworkers specifically assigned for the role. However, understanding of the features and benefits of the scheme for informed decisions was less consistent. Survey respondents suggested enriched information related to eligibility for application (35.2%) and the design of the voucher scheme (32.0%). An enhanced person-centered approach by caseworkers to discuss issues that may be of concern to the elderly people and their carers and to work through the options for better understanding is required to allow informed decisions for participation in the voucher scheme.

The two key elements in the voucher design that influence the participation of the targeted beneficiaries are the “money-following-the-user” principle, which provides greater flexibility and choice according to their needs, and the principle that “users pay in accordance with affordability”. The features in the voucher design that relate to the first element of flexibility and choice according to need are (i) additional subsidized residential care places and private homes that have improved space and manpower standards for provision, (ii) a standard service package that generally reflects the needs of elderly people, (iii) receiving residential care in a shorter period of time for elderly people with more urgent needs, (iv) a 6-month trial period for elderly people to adapt and evaluate the experience with the option of withdrawing from the voucher scheme and reinstatement in the central waiting list for placement, and (v) the ability to switch providers in the scheme. The “money-following-the-user” approach of the voucher scheme allowed the voucher users to choose nonsubsidized places in homes for elderly people, leading to a reduction in waiting time on the central waiting list. Most of the users in the survey (89.0%) agreed that the voucher reduced the waiting time for residential care services, providing an alternative pathway for them to choose nonsubsidized places. A total of 45.8% of users and their carers answered that the reason for participating in the voucher scheme was an urgent need for residential care. A total of 78.1% of users and their carers thought the voucher scheme could help to increase choice and flexibility. However, we found that their choices were limited by the supply and availability of preferred beds in the participating homes, as also reflected in a study in England that examined the low uptake of direct payments in residential care [35, 36]. The findings of the focus group discussions showed that elderly people and their carers preferred subvented and contracted homes, which are thought to be of higher quality; this finding echoes those of studies of He & Chou [4] and Chi [16]. Since most vacancies are in private homes (79.8%) [32], there is a mismatch between preferences and the supply of places. Service quality is a critical consideration for elderly people’s decision to enroll in the scheme. With regard to users’ choice of private homes, users particularly agreed that the enhanced space and manpower standards for participating residential care homes would improve the quality. There was also agreement that giving users purchasing power would encourage homes for elderly people to improve their quality to remain competitive because users had an option to switch homes if they were not satisfied. However, due to gaps in service quality, monitoring is also a critical component in residential care services so that users are willing to choose private homes. Quality could be enhanced by instituting different measures, such as professional codes, training for practitioners in the settings of home for elderly people, and the use of accreditation [7]. The casework services in the voucher scheme could also improve the quality of homes for elderly people since caseworkers follow up with users after they are admitted to these homes. However, manpower implications and long-term sustainability for such casework services when the number of voucher users increases need to be considered. Currently, the voucher scheme provides a standard service package, and 87.8% of users agreed that it met their needs. However, more nonusers disagreed (21%), and there were questions about whether service packages for elderly people could be based on their individualized needs rather than standard packages, including more intensive rehabilitation and escort services to meet their needs arising from deteriorating health conditions. There was also a suggestion to include counseling and psychosocial services in the voucher scheme. This idea is supported by Theurer et al.’s study indicating that interventions that improve elderly people’s social identity and enhance reciprocal relationships are pivotal to address and advance the quality of psychosocial care in the spectrum of residential care [37].

The other critical element of the residential care service voucher scheme is the “users pay in accordance with affordability” (shared responsibility) principle, which is reflected in the copayment mechanism and top-up arrangement. Only 57.0% of nonusers agreed that the copayment mechanism was suitable. Many carers in the focus groups stated that if the copayment was higher than the non-means-tested standard copayment fees charged for all government-funded places in different types of homes allocated from the central waiting list, elderly people would have less incentive to participate. The copayment mechanism needs to be considered in the context of the different residential care service schemes available and the affordability by elderly people and their families. The long-term care cash allowance in Austria is non-means-tested with the levels of allowance solely determined by the assessment of health conditions and needs [38]. The amount of the copayment in Spain’s voucher scheme is determined by the government based on the income of both elderly people and their children but is not fixed at different copayment levels [39]. A flexible value with a maximum limit has the advantage of controlling home fees under a certain price level, but it might cause homes to charge the highest permitted fees under the maximum limit [39]. In Hong Kong, this means-tested copayment ($204–1532) in the voucher scheme could be higher than the non-means-tested all-inclusive fee of $263 for subsidized places on the central waiting list, which might deter some potential users from joining. The majority of current voucher users (87.0%) do not need a copayment since they are fully subsidized by the government (level 0) from the SWD administrative database in 2021. This might lead to an unintended evolution of the voucher scheme where only those who have urgent needs and face difficulties in continuing to be on the central waiting list to join the voucher scheme are less well off, whereas those who are better off have the option of remaining on the central waiting list until a subsidized place is available with the non-means-tested provision. External influences, such as different residential care policies or initiatives, influence the effectiveness of voucher schemes. An alignment of the pricing of different government schemes for residential care homes, the corresponding services provided and their quality needs to be considered in the design of a voucher scheme to allow more incentives for participation. The SWD administrative database shows that 36.9% of voucher users made top-up payments mainly for upgrading dormitories and for rehabilitation or nursing services. A total of 66.8% of voucher users from the survey were willing to make top-up payments to purchase enhanced or value-added services in addition to the standard service package. Transparency of items and fees related to top-up payments is critical to ensure that voucher users make informed choices and decisions about top-up services.

There is no single design of a voucher model that can be applicable to different countries, societies and contexts. The design and implementation of the voucher scheme must be congruent with the objectives and context of the long-term care system and the prevalent social conditions. The pilot for residential care service vouchers in Hong Kong is an innovative policy initiative to address the challenges in the financing and provision of residential care to provide additional care for elderly people in need under the principles of “money-following-the-user” and “users pay in accordance with affordability”. The evaluation of this voucher scheme has encouraged the government to regularize the voucher scheme to a recurrent basis in 2022–2023. In the long run, residential care service vouchers should also be in synergy with community care services vouchers to meet the various long-term care needs of elderly people, similar to Austria’s experience of providing cash allowances in the form of vouchers for elderly people to purchase both institutional and community care services [38, 40]. The promotion of a continuum of care with aging in place and support by residential care services is important for synergy. The integrated use of residential and community care services vouchers could help elderly people and their carers seek appropriate long-term care services in an affordable and adequate way [41].

The major limitation in this study is the limited proportion of nonusers in the survey due to their relatively lower willingness to participate in the study, which can be explained by their relatively limited knowledge of the voucher scheme. The interviewers were trained to encourage participation by convincing potential respondents of the confidentiality of information provided, reiterating the importance of their opinions, and offering incentives. Research from the perspective of homes for elderly people could provide more insight into how to improve the design and operation of the voucher scheme, which is another part of the evaluation of the voucher scheme commissioned by the government. Nevertheless, the findings and themes were consistent across participants in the quantitative and qualitative studies.

## Conclusion

This study demonstrates how the design of a voucher scheme affects its take-up by targeted beneficiaries. When it is implemented in a long-term care system, it must consider the congruence with existing policies in long-term care provision and financing. The voucher scheme in Hong Kong, as a demand-side mechanism, has been able to generate the utilization of nonsubsidized places in homes for the elderly that were underutilized, but its effectiveness is limited by inadequate knowledge and understanding of the voucher scheme and the choice of residential care places. Giving purchasing power and the choice of providers to beneficiaries has the potential to enhance the quality of services, which will contribute to meeting objectives. A monitoring and quality assurance mechanism is necessary to ensure quality. The study findings carry significant implications for long-term care policies and provide insight into the key features of the voucher scheme for residential care services and how to best design and implement a voucher scheme for elderly people in the context of policy objectives and a long-term care policy.

## Data Availability

The dataset generated and/or analyzed during the current study are available from corresponding author based on reasonable request.
